# Training of NANDA‐I Nursing Diagnoses (NDs), Nursing Interventions Classification (NIC) and Nursing Outcomes Classification (NOC), in Psychiatric Wards: A randomized controlled trial

**DOI:** 10.1002/nop2.244

**Published:** 2019-03-04

**Authors:** Taraneh Taghavi Larijani, Babak Saatchi

**Affiliations:** ^1^ Psychiatric Nursing Department, School of Nursing and Midwifery Tehran University of Medical Sciences Tehran Iran

**Keywords:** NANDA‐I Nursing Diagnosis, nursing care, Nursing Interventions Classification and Nursing Outcomes Classification Terminologies, patient safety, psychiatric nursing

## Abstract

**Aim:**

To assess the effect of Training NANDA‐I Nursing Diagnoses, Nursing Interventions Classification and Nursing Outcomes Classification (The NNN system), on the nursing care related to the patient safety, in psychiatric wards.

**Method:**

In a randomized controlled trial, 80 nurses were selected randomly and assigned into two, Control and Experimental, groups. Nurses documented reports, reviewed and analysed in terms of using the NNN system. The intervention of the study was the training of the NNN system, based on recognition of the signs, symptoms and aetiology of the important phenomena in the psychiatric wards.

**Results:**

The Control Group used the NNN system (*N* = 34), both before and after the intervention, while the experimental group usage increased from (*N* = 26)–(*N* = 434). Therefore, the NNN system training, can improve the nursing care related to the patient safety in psychiatric wards.

## 1 INTRODUCTION

According to the statistics, a great deal of research done among the nursing classifications belongs to NANDA‐I Nursing Diagnoses. This classification is also the most widely used international classification in nursing (Müller‐Staub, Needham, Odenbreit, Ann Lavin, & Van Achterberg, 2007).

Nursing Diagnosis (ND) is a clinical judgement about a person, family or community, to potential or actual health problems and life processes. Nursing diagnosis is essential for choosing the nursing interventions in order to achieve the expected outcomes (Herdman & Kamitsuru, 2007). Nursing Interventions Classification (NIC) are the nursing treatments that are performed by the nurses, based on the clinical judgement and knowledge, to improve the outcomes in the patients. NIC include the therapeutic interventions, which are directly implemented on the patient (Bulechek & McCloskey, 1996). Nursing Outcomes Classification (NOC) are changes in health status of the patients, according to the NIC that were done (Maas, Johnson, & Moorhead, 1996). NANDA‐I Nursing Diagnoses (NDs), NIC and NOC are also known as the NNN system. In fact, these components are three phases that related together like "The Loops of a Chain". In the first step, a nurse identifies the patient's problems, with signs and symptoms that are seen in the patient, and this is the stage of identifying Diagnosis, or Diagnoses. In the next step, the nurse plans and selects the appropriate interventions for the patient, that is called Nursing Interventions, and in the final step, determines the expected outcomes, so‐called Nursing Outcomes, in regard to the implemented interventions, and then evaluates the whole process, "The NNN system".

Importance of using the NNN system and its components can be assessed in different ways. Practicality of Nursing Process is in line with the advancement of NANDA‐I NDs and NIC, which generally lead to systematization of the nursing care (Müller‐Staub, Lavin, Needham, & Van Achterberg, 2006). Using proper Nursing Diagnoses is the prerequisite for choosing the appropriate interventions to reach the expected Nursing Outcomes. Therefore, coherence in use of the diagnoses, interventions and outcomes classifications is crucial (Thomé, Centena, Behenck, Marini, & Heldt, 2014). Employing the nursing diagnoses, interventions and outcomes also improves the quality of nursing documents. It is suggested to use the NNN system in documentation of the nursing reports, due to the fact that using standard nursing language in the reports provides an opportunity to evaluate the nursing outcomes, which is essential for assessing the impact and the quality of provided nursing care (Müller‐Staub, 2009). With regard to the studies conducted on this subject, it is found that NANDA‐I NDs cover the important phenomena in psychiatric wards (Frauenfelder, Müller‐Staub, Needham, & van Achterberg, 2011). ND and NIC are also cover most of the nursing care delivered in these settings (Frauenfelder, van Achterberg, & Müller‐Staub, 2018a, 2018b; Thomé et al., 2014).

## 2 BACKGROUND

The existing research indicates that current classifications have been developed; despite most nurses have educated for using these standard nursing languages (Müller‐Staub, 2009), there are some deficiencies in stating the accurate nursing diagnoses and choosing the appropriate interventions and outcomes (Bartholomeyczik & Morgenstern, 2004; Müller‐Staub, 2009). Evaluation of the ND and NIC in the psychiatric settings also represents the lack of information publishing associated with these classifications (Thomé et al., 2014).

Majority of the nurses have connected with ND during their academic education, however, their knowledge and skills were not transmitted into their daily practice and NANDA‐I NDs are not used in these settings (Frauenfelder, van Achterberg, Needham, & Müller Staub, 2016).

Psychiatric nursing is a part of nursing profession that addresses needs of the patients. Nurses in this field learn how to deal with the patients’ psychological needs and their challenging behaviours, as well as building a therapeutic relationship with them, and administering their medications (Stuart; Zarea, Nikbakht‐nasrabadi, Abbaszadeh, & Mohammadpour, 2013). One of the main aspects of the nursing care in these settings is patient safety, due to the presence of the violent behaviours in the patients with psychiatric disorders, suicide and their unstable behaviours (Cleary, Edwards, & Meehan, 1999; Zarea et al., 2013), so these factors could clarify the sensitivity and importance of the patient safety in psychiatric settings. Nursing interventions based on the nursing diagnoses are used to prevent, treat illnesses and promote health (Thomé et al., 2014). The components of the NNN system cover the nursing care provided in the psychiatric wards, so this system is applicable in these settings (Frauenfelder et al., 2011; Thomé et al., 2014). Müller‐Staub (2009) stated that only stating the diagnostic titles is not sufficient to identify the patient needs, and aetiology based diagnoses would be better for choosing the effective nursing interventions, so it can probably result in a better outcome. Thus, it is recommended that the educational strategies for nurses, focus on identification of the signs, symptoms and aetiology of the diagnoses (Müller‐Staub, 2009; Müller‐Staub et al., 2006).

The NANDA‐I NDs has different areas for patient needs, named as "Domain". Among these domains, a particular domain is devoted to the Patient Safety, categorized as *Safety/Protection*. This domain has classes such as *Infection, Physical harm, Violence, Environmental hazards, Defensive processes and Thermoregulation*. Several nursing diagnoses in this domain are as follows: risk for other‐related violence, risk for self‐directed violence, self‐mutilation, risk for self‐mutilation and risk for suicide (Herdman & Kamitsuru, 2007). These nursing diagnoses cover the phenomena and concepts that are common in the psychiatric wards. By reviewing the studies in regard with use of the NNN system in the psychiatric wards, there are no conducted studies, on the training of this system, and in particular, the safety domain. So accordingly, due to the lack of training programmes for the healthcare system staff, and particularly the nurses, as the central part of this workforce, training of the NNN system on the nursing care related to patient safety in psychiatric wards could be important.

## 3 RESEARCH AIM AND QUESTIONS

The aim of the study was to assess the effect of Training NANDA‐I NDs, NIC and NOC on the nursing care related to the patient safety in psychiatric wards.

The following research questions were addressed as follows:

- The frequency of the NNN system usage, in the NANDA‐I *Safety/Protection* domain.
- The effect of NNN system training on the nursing care related to the patient safety.

## 4 METHODOLOGY

### 4.1 Design

This study was a Randomized Clinical Trial (RCT). Documented nursing reports were reviewed in terms of using the NNN system related to the NANDA‐I *Safety/Protection* domain. This study was approved by the Joint Committee of Ethics, of Nursing and Midwifery school with the School of Rehabilitation, Tehran University of Medical Sciences (TUMS) with IR.TUMS.FNM.REC.1396.2697 code. The permission for data collection was obtained from the School of Nursing and Midwifery education office and the Nursing Office of Roozbeh Hospital. According to the written commitment to the Roozbeh Hospital's nursing office, the patients’ personal information has not been collected, and only the nursing documented reports, in terms of using the NDs, NIC and NOC were gathered by the research team. The study was also registered at the Iranian Center for Clinical Trials (IRCT) with the code of; IRCT20170917036237N2.

### 4.2 Setting

The study was performed in *Roozbeh Hospital*, a psychiatric centre with 204 active beds and almost 190 nurses (patient ratio of 1.07), located in Tehran, Iran. This hospital is affiliated with TUMS and includes different wards such as men, women, children, emergency, addiction and the inpatient clinics. It is one of the first and leading psychiatric centres in Iran.

### 4.3 Participants and data collection

The study participants were the nurses working in the Roozbeh Hospital. Eighty of them were selected and assigned into two, Control and Experimental, groups randomly, by permuted‐block randomization. With regard to the aims of the study, our goal was to examine the impact of training among the group that received the intervention (The Experimental group), with the group that did not receive any training (The Control Group). All the nurses were asked for written informed consent regarding participation in the study.

The data were collected by the researcher in a 9‐month period. The nursing reports were selected randomly, in a way that one nurse was chosen, and three documented reports of that nurse were assessed in respect to the frequency of the NNN system usage. The time interval between the pre‐test and post‐test in this study was 12 weeks. Moreover, due to the similarity of the research site and the possibility of association between the two groups, the pre‐test and the post‐test were done in the Control Group at first, and then, these steps were done in the experimental group afterwards.

The inclusion criteria involve the following: written and informed consent to participate in the research, having at least a bachelor's degree in Nursing and working in the psychiatric hospital as a clinical nurse. The exclusion criteria include the following: refuse to continue participating in the study, transfer from the psychiatric hospital to another centre during the study period. Data were collected with two questionnaires. One questionnaire examines the demographic and occupational information, and another one examines the frequency of using NDs, NIC and NOC in the nursing documents.

### 4.4 Intervention of the study

Intervention of the study was the training of NANDA‐I NDs, NIC and NOC related to the *Safety/Protection* domain, during the four sessions, each lasted for 4 hr and in a 2‐month period in the Roozbeh Hospital. This training was educated by the researcher. *Safety/Protection* domain has NDs in six classes such as *Infection, Physical harm, Violence, Environmental hazards, Defensive processes and Thermoregulation*. With each ND, related NIC and NOC were also taught. By reviewing the similar studies (Frauenfelder et al., 2018a, 2018b), one of the best ways to examine the diagnoses, interventions and nursing outcomes used by the nurses working in psychiatric setting is to review the nursing documents. Training of the NNN system in this study focused on training of the signs, symptoms and recognition of the nursing diagnoses aetiology. In this regard, the defining characteristics and risk factors of every NDs were included in the training to select the appropriate interventions and consequently, identification of short, medium and long‐term outcomes.

### 4.5 Data analysis

**Figure 1:**
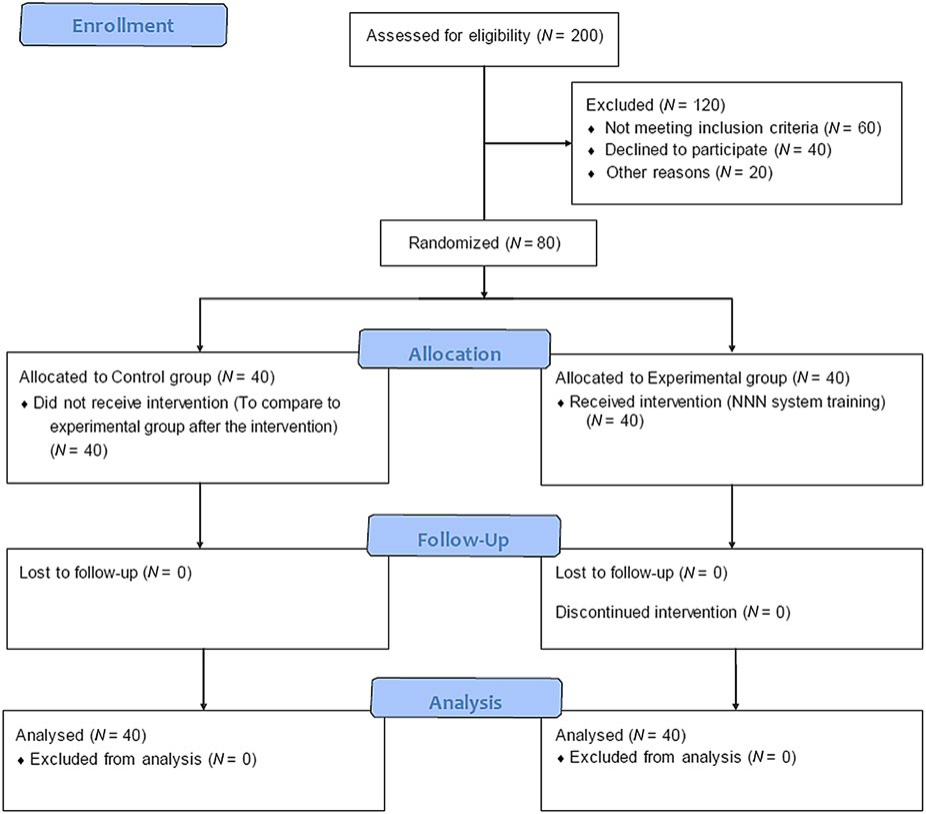
CONSORT flow diagram

Descriptive statistics were applied to calculate the frequencies of identified NNN system use in the nursing documents. Statistical analysis was performed using *IBM SPSS* *Statistics 23* software (IBM Corp., Armonk, NY, USA). The Pearson chi‐square, independent‐samples *t* test and paired‐sample *t* test were adapted to detect the frequency and absolute distribution of the participants age, work experience, gender, level of education, working department and comparison between the NDs, NIC and NOC used in two groups' nursing reports, before and after the intervention. The level of significance was set at 0.05. Steps of the Methodology are summarized in the Figure 1. CONSORT flow diagram.

## 5 RESULTS

Most of the research samples were <30 years old and had a work experience of 1–5 years in psychiatric settings. Also, most of them were female. Bachelor's degree in nursing is the majority in the level of education. Samples were also more active in the men, women, emergency, paediatric departments, respectively. Fifty‐two samples had already participated in the similar training courses before this study. More than 95% of the samples agreed with the “Use of the NDs as a Standard Nursing Language” and nearly 70% of them considered the “inadequate training” as the main barrier to not using the NDs in psychiatric wards. Also, there was an option for the participants to add comments for the existed barriers they think that it might prevent using the NDs. The overall demographic and occupational data of the participants are shown in Table 1.

**Table 1:** Demographic and occupational data of the participants

Category		% (N)
Age	<30	60% (48)
	30–50	40% (32)
Gender	Female	80% (64)
	Male	20% (16)
Level of education	Bachelor's degree	81.25% (65)
	Master's degree	18.75% (15)
Work experience in psychiatric settings	1–10 years	82.5% (66)
	More than 10 years	17.5% (14)
Working department	Men	42.5% (34)
	Women	23.75% (19)
	Paediatric	11.25% (9)
	Emergency	21.25% (17)
	Other departments	1.25% (1)
Participation in the similar training courses	Yes	65% (52)
	No	35% (28)
Agree or Disagree, with the use of nursing diagnosis as a standard nursing language	Agree	95% (76)
	Disagree	5% (14)
Main barrier to not using the Nursing Diagnoses (NDs) in psychiatric wards	Inadequate training	96.25% (77)
	High number of patients	3.75% (3)

The findings from using NDs, NIC and NOC (NNN system) before the intervention indicate a low‐level use of this system, in both control and experimental groups. In the Control Group, 20 ND and 14 NIC were used in different classes before the intervention, while after the intervention, 21 ND and 13 NIC were used. NOC were not used, before and after the intervention, nevertheless (Figure 1).

In the experimental group, the frequency of using the NNN system was increased considerably, so that the number of the NDs used increased from 22–202. The frequency of the used NIC reached from 4–144 times and NOC 0–88. After the intervention, the violence class received the highest number of NDs, NIC and NOC use. Subsequently, classes of physical harm, infection, thermoregulation and environmental hazards were the most commonly used classes in the NNN system, respectively. Defensive process class was not used in any of the control and experimental groups, before and after the intervention.

Comparing the mean amounts of the NNN system use, in the experimental group, before and after the intervention, all the classes in the *Safety/Protection* domain, showed a significant increase, except the “defensive processes” class. Paired‐sample *t* test showed a significant difference (*p* < 0.05) between the means before and after the intervention, in the infection, physical harm, violence, environmental hazards and thermoregulation classes in the NNN system use. Frequency of the classes used between NDs, NIC and NOC in control and experimental groups before and after the intervention is shown in Table 2.

**Table 2:** Frequency of used classes ND, interventions and outcomes in control and experimental groups before and after intervention

Classes	The NNN system use in the Control Group (before and after the intervention)		The NNN used
Infection	No NNN system use before and after the intervention		
Physical harm	ND	Before (*N* = 13)	Risk for falls
		After (*N* = 11)	
Violence	ND	Before (*N* = 7) After (*N* = 10)	Risk for self‐mutilation Self‐mutilation Risk for other‐directed violence Risk for suicide
	NIC	Before (*N* = 6) After (*N* = 6)	Behaviour management: self‐harm Surveillance Suicide prevention Seclusion
Environmental hazards	NIC	Before (*N* = 1) After (*N* = 0)	Surveillance
Defensive processes	No NNN system use before and after the intervention		
Thermoregulation	No NNN system use before and after the intervention		

Classes	The NNN system use in the Experimental group (before and after the intervention)		The NNN used
Infection	ND	Before (*N* = 0) After (*N* = 33)	Risk for infection
	NIC	Before (*N* = 0) After (*N* = 20)	Health education Immunization/Vaccination management Infection control
	NOC	Before (*N* = 0) After (*N* = 9)	Health‐promoting behaviour Health‐seeking behaviour
Physical harm	ND	Before (*N* = 19) After (*N* = 24)	Risk for falls
	NIC	Before (*N* = 0) After (*N* = 24)	Fall prevention
	NOC	Before (*N* = 0) After (*N* = 15)	Fall prevention behaviour
Violence	ND	Before (*N* = 3) After (*N* = 105)	Self‐mutilation Risk for self‐mutilation Risk for self‐directed violence Risk for suicide
	NIC	Before (*N* = 4) After (*N* = 74)	Surveillance: safety Anxiety reduction Behaviour management: self‐harm Counselling Documentation Impulse control training Limit setting Relaxation therapy Risk identification Socialization enhancement Suicide prevention Presence Physical restraint Restraint
	NOC	Before (*N* = 0) After (*N* = 52)	Anxiety self‐control Coping Hope Risk control Risk detection Will to live Social interaction skills Suicide self‐restraint
Environmental hazards	ND	Before (*N* = 0) After (*N* = 9)	Risk for poisoning
	NIC	Before (*N* = 0)	Medication management
		After (*N* = 7)	Medication Reconciliation
	NOC	Before (*N* = 0) After (*N* = 3)	Medication response
Defensive processes	No use from NNN system before and after the intervention		
Thermoregulation	ND	Before (*N* = 0) After (*N* = 31)	Risk for imbalanced body temperature Hypothermia Ineffective thermoregulation
	NIC	Before (*N* = 0) After (*N* = 19)	Temperature regulation
	NOC	Before (*N* = 0) After (*N* = 9)	Thermoregulation

Note

ND: Nursing Diagnosis; NIC: Nursing Interventions Classification; NOC: Nursing Outcomes Classification.

## 6 DISCUSSION

The present study investigated the frequency of using the NNN system in the nursing care related to the patient safety, in the context of psychiatric wards. The results indicate a low level use of the NANDA‐I Nursing Diagnoses (NDs), NIC and NOC (the NNN system) in psychiatric wards, before performing the intervention. Regardless of 65% of the nurses that are participated in the similar training courses before carrying out this study. There was a limitation existed in this study, and that was not being able to observe the care provided by the nurses in different wards, in a one‐to‐one observation.

Among all of the nursing documents, in both control and experimental groups, before the intervention, only 42 ND and 18 NIC were used totally, while no NOC used among them. The low amount of using the NNN system could be analysed from the several aspects. As noted by Müller‐Staub et al. (2006), merely stating the diagnostic expressions is not sufficient to identify patients’ needs and aetiology‐based diagnosis could lead to selection of the effective nursing interventions and as a result, better outcomes. Therefore, nursing education planning should focus on improving the accuracy of ND and diagnostic reasoning, based on identification of the signs and symptoms and the diagnostic aetiology (Müller‐Staub, 2009; Müller‐Staub et al., 2006). So, in fact, one of the reasons for the low level of NNN use in this study is the deficiency in accurate use of the NDs. Due to the fact that all three components of the NNN system are interlinked, inappropriate employment of its first step, which is the ND, could lead to the inaccurate use of the whole system. So, precise NDs are essential for choosing the NIC based on the diagnoses lead to the desired NOC (Müller‐Staub, 2009). In this study, aetiology‐based diagnoses were educated to the nurses and this resulted in the increase in the NNN system use.

Another reason for the lack of NNN system use in this study may be the insufficient knowledge of the nursing taxonomies and classifications among the nurses. As Frauenfelder et al. (2016) stated, although most nurses working in psychiatric departments are associated with nursing diagnoses during their academic education, their knowledge and skills have not been transmitted to routine care and the NANDA‐I classification is not employed in these settings (Frauenfelder et al., 2016). Despite the NANDA‐I classification is the only standard nursing language that is used in nursing reports, in relation to the patient problems (Frauenfelder et al., 2016), nursing managers and nurses working in psychiatric settings often know little about the criteria that can be expected from the nursing diagnostic classifications (Müller‐Staub, Lavin, Needham, & van Achterberg, 2007). Another point is the importance of using the NNN system in psychiatric settings. As mentioned before, many deficiencies were found in use of the NANDA‐I ND and NIC taxonomies in the field of mental health (Thomé et al., 2014), whereas the nursing interventions could be effectively implemented in mental health settings, to prevent, treat diseases, or promote health, and lead to improve of the health outcomes (Dochterman & Bulechek, 2008; Thomé et al., 2014).

The importance of reporting and the documentation of nursing care are also the another important factor which needed to be considered. Nurses are required to describe, record and evaluate their participation in the health care. In many countries, nursing reports are a part of patients’ health history and health legislations require the writing of medical and nursing care. Patient's problems that are identified, implemented nursing interventions and evaluation of the care provided should be documented. Thus, the nursing documents not only report and compare, but also ensure and improve the quality of the nursing care. For these reasons, the classifications that lead to the standardization of nursing language should be implemented in the nursing (Müller‐Staub, 2009). Considering the significance of documentation in the nursing care, the nurses may take a part in taking care of the patient, but in the meantime they could be unfamiliar with the effective documentation of the care they are provided. Studies have shown that quality of the documented nursing reports is improved by implementation of the NNN system (Müller‐Staub, 2009), in agreement with the intervention that was performed in this study.

Given the fact that this study addresses the patient safety, which is an important aspect of the nursing care implemented in psychiatric settings, low use of the NNN system in these settings can be critical, because the patient safety is an important and integral part of the nursing care. As Slemon, Jenkins, and Bungay (2017) states in terms of the patient safety, in mental health setting: “the safety issue is considered to be the highest value beyond a consideration or goal” (Slemon et al., 2017). In other words, the first goal of psychiatry is to keep patients and other people safe (Nijman et al., 2011). Frauenfelder et al. (2018a, 2018b) concluded in their study of ND related to psychiatric wards that the most NDs were used in copping/stress tolerance, safety and health promotion domains. In the safety domain, self‐mutilation, risk for self‐mutilation, risk for suicide, risk of self‐directed violence and the risk of other‐directed violence were the large number of diagnoses (Frauenfelder et al., 2018a, 2018b). So these findings are consistent with the results of the present study. In addition, the variety in use of the NDs, NIC and NOC was another remarkable result of this study. Among the classes of Safety/Protection domain, the *Violence* was the mostly used class, and then, physical harm, infection, thermoregulation and environmental hazards were the most frequently used classes. Therefore, it can be concluded that one of the most important aspects of the nursing care related to the patient safety in psychiatric wards is the violence and its related concepts. So, training of the NNN system in psychiatric wards, should better focus on teaching concepts related to the violence. Moreover, the class of “defensive processes” that includes “risk of allergic response” ND was not used to any extent, so it needs to be investigated.

## 7 CONCLUSION

NANDA‐I Nursing Diagnoses (NDs), NIC and NOC or the NNN system promote the nursing care related to the patient safety in psychiatric wards. The NNN system training enhanced the frequency of using NDs, NIC and NOC, as well as the variety of used components. With regard to the high use of the violence class, it is recommended to focus on the nursing care related to the violence and the concepts connected to it. More studies could be conducted on the class of violence, as well as other NANDA‐I classification domains in psychiatric wards.

## ETHICAL STATEMENT

This study was approved by the Joint Committee of Ethics of Nursing and Midwifery school with the School of Rehabilitation, Tehran University of Medical Sciences (TUMS) IR.TUMS.FNM.REC.1396.2697 code.

## CONFLICT OF INTEREST

No conflict of interest has been declared.
